# Topography-Guided Transepithelial Accelerated Corneal Collagen Cross-Linking for Low Refractive Error Correction in Keratoconus Treatment: A Pilot Study

**DOI:** 10.3389/fbioe.2022.830776

**Published:** 2022-02-23

**Authors:** Ling Sun, Xiaoyu Zhang, Mi Tian, Yang Shen, Jing Zhao, Xingtao Zhou

**Affiliations:** ^1^ Department of Ophthalmology, Eye and ENT Hospital, Fudan University, NHC Key Laboratory of Myopia (Fudan University), Laboratory of Myopia, Chinese Academy of Medical Sciences, Shanghai, China; ^2^ Shanghai Research Center of Ophthalmology and Optometry, Shanghai, China

**Keywords:** transepithelial accelerated corneal collagen cross-linking, refractive error, keratoconus, pilot study, topography guided

## Abstract

**Purpose:** To investigate the safety and efficacy of topography-guided transepithelial accelerated corneal collagen cross-linking for low refractive error correction in patients with keratoconus.

**Methods:** This was a prospective self-controlled study. Eighteen patients (18 eyes) were enrolled and assessed at 6 visits (pre-operation, 1 w, 1 month, 3 months, 6 months, and 1 year postoperatively). The examination at every visit included analysis of uncorrected visual acuity (UCVA), best-corrected visual acuity (BCVA), corneal topography, and corneal endothelial cell counts. Data are expressed as mean ± standard deviation (SD). The *p*-value was determined using repeated-measures analysis of variance.

**Results:** No complications occurred in any eye during 1 year follow-up period. At each visit after the operation, the corneal K values and spherical equivalent (SE) were reduced, while the visual acuity values were increased compared with those preoperatively, although these results were not statistically significant (*p* > 0.05). UCVA of nearly 1/3 of the patients was enhanced by at least 3 lines at each follow-up visit. During the whole follow-up, corneal endothelial cell counts were stable (*p* > 0.05). Regarding topography, part of the corneal cone was flattened after the operation.

**Conclusion:** Topography-guided transepithelial-accelerated corneal collagen cross-linking is safe and may correct low refractive error in keratoconus treatment. Further studies and improvements are required in this regard.

## Introduction

Keratoconus is a chronic ectatic corneal disease that leads to progressive corneal thinning and irregular astigmatism. A substantial number of keratoconus patients suffer severe visual impairment, which may severely disrupt their lives. These individuals usually use spectacles and rigid gas-permeable contact lenses to improve their visual acuity; however, these instruments are inconvenient and can damage the cornea when keratoconus progresses.

Currently, corneal collagen crosslinking (CXL) is accepted by ophthalmologists as a technique that changes the biomechanics of the cornea and delays or prevents the development of corneal ectasia (Wollensak et al., 2003; Vinciguerra et al., 2013). Meanwhile, many studies have shown that patient visual acuity improves, while corneal curvature flattens after the operation (Kanellopoulos, 2012; Wittig-Silva et al., 2014; Chow et al., 2015). Therefore, some scientists have hypothesized that corneal collagen cross-linking may be used in the treatment of low refractive error correction if this technique can be modified to obtain more obvious corneal flattening. This new technique could benefit patients who are unsuitable for corneal ablation. Roy et al. (Sinha Roy and Dupps, 2011a) found that corneal cone irradiation resulted in greater reduction in curvature than full corneal collagen cross-linking patterns. Based on this theory, the concept of topography-guided corneal collagen cross-linking was proposed, and a related system was developed (KXL II device, Avedro Inc., Waltham, MA, United States).

To date, several case reports of topography-guided transepithelial corneal collagen cross-linking have been published, and the results have shown that patients demonstrated improved vision and decreased corneal curvature (Kanellopoulos, 2014; Kanellopoulos et al., 2014). In this prospective study, we investigated the feasibility of topography-guided transepithelial-accelerated corneal collagen cross-linking (TG-CXL) for low refractive error correction in patients with keratoconus.

## Materials and Methods

### Subjects

This was a prospective self-controlled study. Eighteen patients (18 eyes) were recruited from the Department of Ophthalmology of the Fudan University EENT Hospital. The enrolled eyes were diagnosed with keratoconus and were kept stable for 12 months. The stability of keratoconus was defined as less than 0.5 diopter(D) increase in maximum keratometry, manifest cylinder, manifest refraction spherical equivalent, or no loss or loss of one line of corrected distance visual acuity over the previous 12 months (Kamiya et al., 2015; Doroodgar et al., 2017). The diagnosis of keratoconus was based on corneal topographic data and stromal thinning.

Inclusion criteria were the following: patients aged at least 18 years;

CDVA of 20/80 or better; SE 0 to −3.00 D; minimal corneal thickness 475 micron or better; clear cornea without visible scar on slit-lamp examination.

Exclusion criteria were previous ocular trauma or surgery, other ocular disease or systemic disease that may affect the cornea, and vitamin C (ascorbic acid) supplementation within 1 week of the cross-linking treatment.

The Amsler–Krumeich (AK) classification of keratoconus was used in this study (Rabinowitz, 1998-; Kamiya et al., 2014). According to this classification, 10 eyes were stage I, 5 eyes were stage II, and 3 eyes were stage III. Routine preoperative examinations were performed in order to exclude patients with contraindications. All the patients fully understood the procedure and provided signed informed consent. This study was approved by the Ethics Committee of the Fudan University EENT Hospital Review Board and followed the tenets of the Declaration of Helsinki.

At each visit, the following parameters were assessed: corrected distance visual acuity (CDVA), slit-lamp biomicroscopy, corneal topography, optical tomography, pachymetry with the Pentacam (Oculus, Germany), and endothelial biomicroscopy (NIDEK, Japan). All the postoperative complications were recorded.

### Surgical Techniques

**FIGURE 1:**
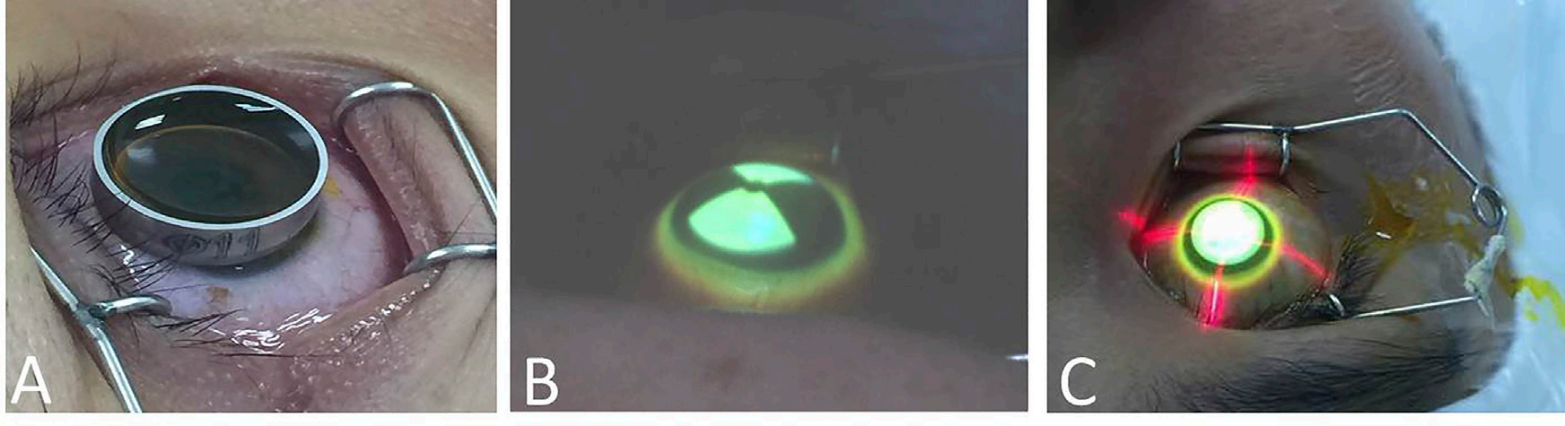
The operation process. **(A)** Application of riboflavin. **(B)** According to the corneal topography, UVA was applied in a sector shape. **(C)** According to the corneal topography, UVA was applied in a circular shape.

All surgeries were performed by the same surgeon (ZXT). After topical anesthesia with 4% oxybuprocaine, 0.1% riboflavin (ParaCel, Avedro Inc.) was applied to the cornea for 4 min, followed by 0.25% riboflavin (VibeX Xtra, Avedro Inc.) for 6 min. The cornea was irradiated with UVA light at 45 mW/cm^2^ and pulsed illumination. Treatment profiles included three concentric circular zones centered on maximum posterior elevation to mimic a graded treatment pattern based on the theoretical model proposed by Sinha Roy and Dupps, which suggests that spatial variation of UVA intensity results in the greatest flattening effect (Sinha Roy and Dupps, 2011b). The total UVA dose was 5.4 J when the corneal curvature was no more than 48D, 10 J when it was 48D-51D, and 15 J when it was larger than 51D. The irradiation time and shape were calculated using the KXL II system (Avedro Inc., United States) and corneal topography data (Figure 1). At the end of the surgery, a bandage contact lens (ACUVE OASYS, Johnson & Johnson Inc.,) was placed over the cornea.

### Post-Operative Medication

The postoperative topical medication regimens were identical for each eye: 0.3% levofloxacin four times per day for 3 days, a 0.1% fluorometholone eye drops was underwent 6 times a day and decrease 1 times every 3 days, and a preservative-free tear supplement four times per day for 1 month. The contact lens was removed on postoperative day one.

### Follow-Up

The patients were followed-up at 1 week, 1 month, 3 months, 6 months, and 1 year postoperatively. The examinations included UDVA, CDVA, endothelial cell density, and Pentacam corneal topography to obtain flat K (K1), steep K (K2), mean K (Km), and maximum K (Kmax) values.

### Statistical Analysis

Data are expressed as mean ± standard deviation (SD). The *p*-value was determined using single-factor repeated-measures ANOVA. Statistical significance was set at *p* < 0.05. All statistical analyses were performed using SPSS software (version 16.0, SPSS Inc., Chicago, IL, United States).

## Results

No complications occurred in any of the 18 patients (18 eyes) . After the operation, one patient withdrew for personal reasons. Seventeen patients (17 eyes) were included in the data analysis. The patients’ mean age was 24.88 ± 4.64 years, and the male to female ratio was 9:8. One patient was lost after 6 months follow-up.

**FIGURE 2:**
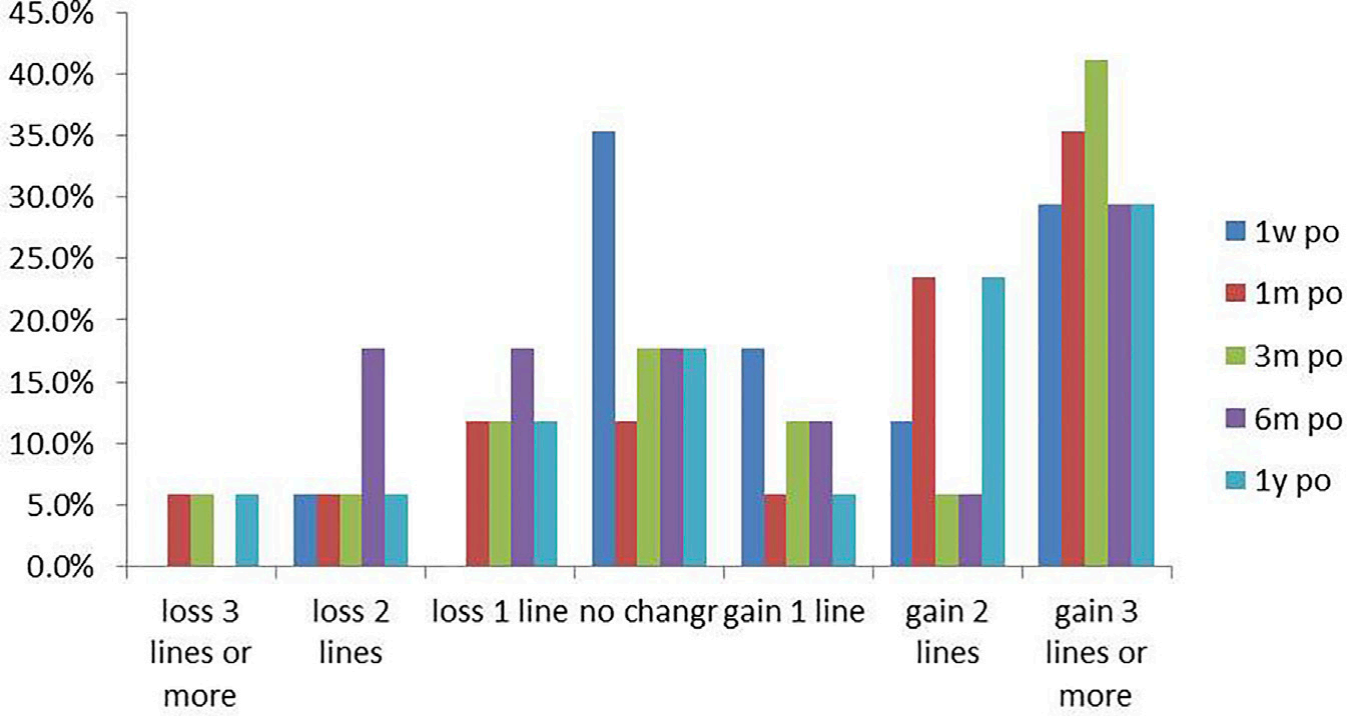
The UCVA of most patients was higher than the pre-operative value, and the UCVA of nearly 1/3 of the patients was enhanced by at least 3 lines at each follow-up visit.

The spherical equivalent (SE), uncorrected distance visual acuity (UDVA), and corrected distance visual acuity (CDVA) at each follow-up visit are shown in Table 1. After the operation, the mean SE at each follow-up point decreased compared to that before the operation. At the 1 year postoperative visit, the average SE decreased by 0.73 ± 0.36 D compared to the preoperative SE. This finding was not statistically significant (*p* > 0.05) . The postoperative UDVA and CDVA both increased, although the differences were not significant (*p* > 0.05) . The UCVA changes of patients were showed in Figure 2, and the UCVA of nearly 1/3 of the patients was enhanced by at least three lines at each follow-up visit (Figure 2). At the last visit, the mean postoperative CDVA and the mean postoperative UCVA were 0.05 ± 0.04 and 0.52 ± 0.08. The safety index (postoperative CDVA/preoperative CDVA ) and efficacy index (postoperative UCVA/preoperative CDVA) were 0.56 ± 1.0 and 5.78 ± 2.0.

**TABLE 1:** Ocular examination results at each follow-up point.

	pre	1w po	1 m po	3 m po	6 m po	1 year po
UDVA (logMAR) (mean ± SD)	0.67 ± 0.09	0.48 ± 0.07	0.48 ± 0.07	0.49 ± 0.07	0.60 ± 0.07	0.52 ± 0.08
CDVA (logMAR) (mean ± SD)	0.09 ± 0.04	0.04 ± 0.03	0.03 ± 0.02	0.02 ± 0.03	0.05 ± 0.04	0.05 ± 0.04
SE(D) (mean ± SD)	−3.62 ± 0.39	−3.30 ± 0.35	−3.23 ± 0.32	−3.07 ± 0.32	−3.01 ± 0.34	−2.90 ± 0.34
Steep K(D) (mean ± SD)	46.44 ± 40.70	46.13 ± 0.65	46.29 ± 0.66	46.19 ± 0.65	46.17 ± 0.64	46.15 ± 0.60
Flat K(D) (mean ± SD)	44.40 ± 0.44	44.14 ± 0.41	44.28 ± 0.44	44.24 ± 0.42	44.19 ± 0.41	44.28 ± 0.44
K max(D) (mean ± SD)	49.13 ± 1.22	48.98 ± 1.22	49.11 ± 1.31	48.76 ± 1.09	48.77 ± 1.14	48.82 ± 1.16
TCT (μm) (mean ± SD)	497.25 ± 5.69	485.69 ± 6.62	489.38 ± 6.29	493.94 ± 6.67	492.25 ± 7.07	492.00 ± 6.86
ECD (mean ± SD)	3,095.01 ± 98.85	3,016.15 ± 122.88	3,047.27 ± 104.38	3,255.69 ± 109.20	3,307.11 ± 116.27	3,269.93 ± 126.46

UDVA uncorrected distant visual acuity; CDVA corrected distance visual acuity; SE spherical equivalent; TCT thinnest corneal thick; ECD endothelial cell density.

**FIGURE 3:**
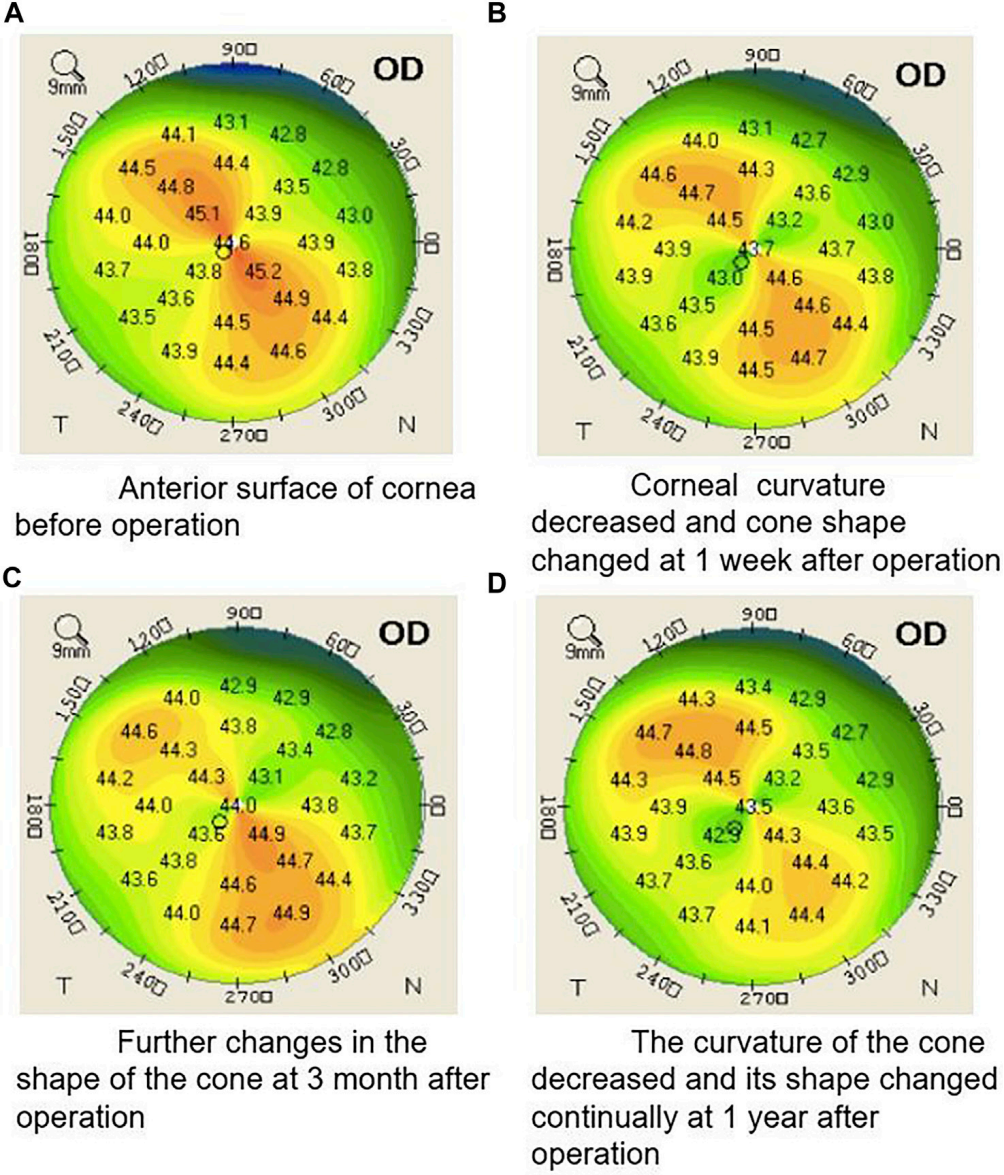
Illustration of a typical case showing the shape of corneal cone changed during the whole follow-up period after the operation **(A)** Anterior surface of cornea before operation. **(B)** Corneal curvature decreased and cone shape changed at 1 week after operation. **(C)** Further changes in the shape of the cone 3 months after operation. **(D)** The curvature of the cone decreased and its shape changed continually at 1 year after operation.

The results of corneal topography examination at each follow-up visit are shown in Table 1. The average postoperative flat K value (K1), steep K value (K2), mean K value (Km), and maximum K value (Kmax) were lower than those determined preoperatively; however, this difference was not statistically significant (*p* > 0.05). The corneal curvatures changed by approximately 0.5D, while the Kmax of the two eyes decreased by 1.5D. Some corneal cones demonstrated flattening in the topographic image during the entire follow-up period (Figure 3).

During the follow-up, corneal endothelial cell counts kept stable (Table 1).

## Discussion

Topography-guided corneal collagen cross-linking is a new technique that has been developed for refractive error correction, the efficacy of which may still be limited; however, for keratoconus patients, the technique is meaningful because it can not only correct refractive error to some extent, but also strengthen the cornea and delay or prevent disease progression. Our study was a prospective self-controlled study and investigated the safety and efficacy of topography-guided transepithelial-accelerated corneal collagen cross-linking for low refractive error correction in keratoconus treatment.

All surgeries were uneventful, and no corneal infection, haze, or other complications occurred at the 1 year follow-up. Moderate corneal epithelial edema was observed on the first postoperative day. At the 1 month postoperative visit, all corneal epithelia had recovered completely. The corneal endothelial cell count did not change significantly at each follow-up visit. These results indicated that topography-guided transepithelial accelerated corneal collagen cross-linking may have good safety comparable to that of general transepithelial-accelerated corneal cross-linking which was showed in author’s previous study (Sun et al., 2018).

At the 1 year postoperative visit, the average SE was decreased by 0.73D, while it declined by more than 1D in 6 eyes (35.3%). The maximum reduction is 1.75D. In 9 patients, UDVA was enhanced by two or more lines. Since an improvement of more than 1D in refraction and 2 lines of vision can be considered clinically significant, these results suggest that topography-guided transepithelial accelerated corneal collagen cross-linking may correct a low degree of refractive error correction in some patients. Although UDVA was lost by 2 lines in 1 eye and 3 lines in 2 eyes compared to preoperative results, We found these eyes were classified to stage II or stage III keratoconus, Kmax was larger than 54D and UDVA was larger than 0.4 before operation. We knew patients’ visual acuity was blurred in severe keratoconus, so UDVA result may fluctuate. Since CDVA was unchanged and K readings were stable, we thought these eyes didn’t get worse after operation.

The Kmax in 2 eyes decreased by 1.5D at the 3 months postoperative visit, while the Kmax value of the other eyes changed by approximately 0.5D throughout the entire follow-up period . The values of K1, K2, and Km decreased slightly after the operation. Although we did not find significantly decreased K values, our results showed that corneal keratometry was stable one 1 year after the operation, which is valuable for keratoconus treatment.

We also found that the decrease in corneal curvature was inconsistent with the decrease in corneal refractive power. For example, the average SE decreased by 0.73D compared to the pre-operation SE at 1 year postoperative follow-up, while the corneal curvatures decreased approximately 0.5D, which was less than changes of SE. Thus, the mechanism of this technology for refractive error correction may include not only corneal flattening, but also other factors, such as remodeling of the corneal epithelium. Further studies are required to confirm this hypothesis.

In our study, the shape of most corneal cones changed after the surgery, where keratometry of part of the corneal cone decreased, while some cone ranges even decreased. Keratometry of most parts was stable outside of the corneal cone. This result was different from that of traditional or accelerated corneal cross-linking without a topography guide, which usually causes a decrease in keratometry both inside and outside the corneal cone. Additionally, the shape and range of the cones did not change. Regarding the mechanism of the technique, topography-guided corneal collagen cross-linking can select different irradiation energies applied to different parts of the cornea according to corneal topography. A greater curvature of the cornea receives more UVA energy; thus, more energy can be provided to the cone area than to the peripheral parts so that the cone can be further flattened. Our results confirm this theory to some extent, and we believe that topography guidance plays a certain role in this operation. Thus, this technique may provide patients with more personalized treatment, selectively reduce the steep portion of the corneal curvature and smooth irregular corneas, and improve visual acuity.

Our results were slightly different from those of published case reports, which demonstrated a more obvious improvement in vision and decreased corneal curvature. For example, in the case report of Kanellopoulos et al. (Kanellopoulos, 2014), the K value of four eyes decreased by 2.3D and 1.44D at 1 week and 6 months postoperatively, respectively. We should note that our study was prospective and enrolled more patients, and thus, the scientificity and reliability should be better compared to the case report. However, we also found that the Kanellopoulos study used a UVA of 12 J, while the average corneal curvature was 44.5D. In the present study, we used much less UVA energy, which might have affected the cross-linking. In Cassagne et al.’s study, CDVA and Kmax improved significantly in the TG-CXL (*p* < .05) (Cassagne et al., 2017). We noted Kmax of patients enrolled in TG-CXL group was 59.23 ± 7.54D, which was much larger than 49.13 ± 1.22D in our study.As Chen et al.’s study showed a higher preoperative maximum K value correlated with greater corneal flattening after epithelium-on CXL (Chen et al., 2016). We hypothesis TG-CXL might be more effective in severe keratoconus treatment. Better results may be obtained using a modified design in future studies.

This study has some limitations. First, the sample size was small, which might have affected the statistical efficacy; therefore, we will continue to enroll more patients for further analysis. Second, the follow-up time may not have been sufficient to evaluate the long-term efficacy. Third, general K values of 9 mm corneal topography were analyzed in this study, which warrants analysis of different regions in future studies with a large sample size as this would yield more detailed information.

In conclusion, we present a new technique that may be applied in keratoconus treatment for low refractive error correction. However, further studies and improvements are needed in topography-guided transepithelial-accelerated corneal collagen cross-linking.

## Data Availability

The original contributions presented in the study are included in the article/Supplementary Material, further inquiries can be directed to the corresponding author.
